# Development of Cre-dependent retrograde trans-multisynaptic tracer based on pseudorabies virus bartha strain

**DOI:** 10.1186/s13041-025-01204-y

**Published:** 2025-04-14

**Authors:** Hu You, Wang Qinghan, Sun Kangyixin, Yang Jia, Xu Fuqiang, Jia Fan

**Affiliations:** 1 Guangdong Provincial Key Laboratory of Brain Connectome and Behavior, CAS Key Laboratory of Brain Connectome and Manipulation, the Brain Cognition and Brain Disease Institute, Shenzhen Institutes of Advance Technology, Translational Research Center for the Nervous System, Chinese Academy of Sciences, Shenzhen-Hong Kong Institute of Brain Science-Shenzhen Fundamental Research Institutions, Shenzhen, 518055 China; 2 NMPA Key Laboratory for Research and Evaluation of Viral Vector Technology in Cell and Gene Therapy Medicinal Products, Key Laboratory of Quality Control Technology for Virus-Based Therapeutics, Shenzhen Key Laboratory of Viral Vectors for Biomedicine, Shenzhen Institutes of Advance Technology, Guangdong Provincial Medical Products Administration, Chinese Academy of Sciences, Shenzhen, 518055 China; 3 Wuhan National Laboratory for Optoelectronics, Huazhong University of Science and Technology, Wuhan, 430074 China; 4 University of Chinese Academy of Sciences, Beijing, 100049 China; 5 Faculty of Life and Health Sciences, Shenzhen University of Advanced Technology, Shenzhen, 518106 China

**Keywords:** Pseudorabies virus, Retrograde trans-multisynaptic tracer, Cre-dependent, PRV676, PRV829

## Abstract

Mapping the neural circuit of a specific neuronal subclass is central to understanding the working mechanism of the brain. Currently, numerous types of transgenic mice expressing Cre recombinase have been engineered and widely used in neuroscience. To map the multilevel inputs into the neural circuit of a specific neuronal subpopulation, a Cre-dependent retrograde trans-multisynaptic tracer must be developed. The vaccine strain of Pseudorabies virus (PRV, Bartha strain) can infect neurons and spread in a retrograde manner in the neural circuit. In this study, we engineered the genome of PRV Bartha strain to prepare two new tracers, PRV676 and PRV829, by replacing the TK gene of PRV with the Cre-dependent expression cassette of the fluorescent protein gene and the TK gene. These two tracers can separately and Cre-dependently express EGFP and mRuby3 and produce progeny viruses in vitro and in vivo, which can help to map the multilevel inputs of a specific neuronal subpopulation expressing Cre. Collectively, our work provides two new tools for neuroscience research.

**Supplementary Information:**

The online version contains supplementary material available at https://doi.org/10.1186/s13041-025-01204-y.

## Introduction

Virus-based tracers have been widely used for mapping the neural circuit of the brain [1–3]. Anterograde trans-synaptic tracers, which include the Vesicular stomatitis virus, Herpes simplex virus type 1 (HSV), Yellow fever virus (YFV), Adenovirus associated virus serotype 1 (AAV1), and Sindbis virus (SINV) [1–3], can depict the output neural circuit of the brain. Retrograde trans-synaptic tracers, including Rabies virus (RV) and Pseudorabies virus (PRV), can be used to map the input neural circuit of the brain [1–3]. In the neuroscience community, it is understood as important to map the neural circuit of a specific neuronal subclass to understand the working mechanism of the brain. Therefore, two tools are needed: (1) various transgenic mice expressing Cre recombinase, which have been engineered and widely used in neuroscience [4–6], and (2) a Cre-dependent trans-synaptic tracer, which is indispensable [1–3]. For mapping the direct input or output neural circuit of a specific neuronal subpopulation expressing Cre, different trans-monosynaptic tracers have been developed by deleting the G (for RV and VSV) [7–10] or TK (for PRV and HSV) [11, 12] genes of these viruses from their genomes, and the proteins encoded by the deleted gene are provided in trans-complementation by a helper virus in neurons expressing Cre. By contrast, only two Cre-dependent trans-multisynaptic tracers have been engineered [13, 14]. However, these tracers have more drawbacks, such as the high cytotoxicity and the low expression level of fluorescence [11, 15, 16]. Therefore, more condition-dependent trans-multisynaptic tracers with favorable properties are still needed.

The PRV Bartha strain can infect neurons and transfer in a retrograde manner in the neural circuit of the CNS and PNS [17, 18]. It has a double-stranded DNA genome, which can be engineered to be a Cre-dependent retrograde trans-multisynaptic tracer and then used to label the multi-level inputs of specific neuronal subpopulations expressing Cre [19]. The PRV TK protein plays a key role in supporting viral replication in the neuron [20], which can be used as the target for preparing a Cre-dependent retrograde trans-multisynaptic tracer. In this study, we developed two new tracers, PRV676 (expressing 3×EGFP) and PRV829 (expressing 3×mRuby3), which Cre-dependently express the fluorescent protein and the TK protein in neurons expressing Cre, after which the progeny virus can spread in a trans-multisynaptic manner in the neural circuit. Together, these two tracers will be beneficial to depicting the multilevel inputs of the specific neuronal subpopulation expressing Cre.

## Materials and methods

### Cell, virus and animal

BHK-21 cells and BHK-21-Cre cells were maintained in Dulbecco’s modified Eagle medium (DMEM) with 10% fetal bovine serum (FBS) and incubated at 37 °C in 5% CO_2_. The Pseudorabies virus (PRV Bartha strain) and the reporter PRV (PRV676 and PRV829) were cultured in DMEM containing 2% FBS at 37 °C in 5% CO_2_. The C57BL/6J mice, DAT-Cre mice, and vGat-Cre mice were housed in cages with free access to food and water under a standard condition of 12 h of light and 12 h of dark. All studies were performed following the National Guidelines for the Care and Use of Laboratory Animals and approved by the Animal Care and Use Committees at the Shenzhen Institutes of Advanced Technology, Chinese Academy of Sciences.

### Construction of the plasmid

To prepare the reporter PRV (PRV676 and PRV829), several plasmids were constructed. To construct the plasmid PS676 **(**left arm-Ubc-DIO-EGFP-P2A-EGFP-F2A-EGFP-T2A-TK-WPRE-hGHpA-CMV-nlsmtagBFP-bGHpA-right arm), several steps were taken, as follows: (1) the left arm covers nucleotides 48,345 to 50,344 in the PRV Bartha strain (Genbank no. JF797217), which were engineered into the digested plasmid 20,298# (Addgene) with MluI and MfeI to prepare the plasmid 20,298-left arm; (2) the fragment of the Ubc promoter was inserted into the digested plasmid 20,298-right arm with MluI and KpnI to prepare the plasmid 20,298-left arm-Ubc; (3) the right arm covers nucleotides 51,300 to 53,299 in the PRV Bartha strain (Genbank no. JF797217), which were engineered into the digested plasmid 20,298-left arm-Ubc with BstEII and RsrII to prepare the plasmid 20,298-left arm-Ubc-right arm; (4) the expression cassette of CMV-nlsmtagBFP-bGHpA was inserted into the digested plasmid 20,298-left arm-Ubc-right arm with BstEII and RsrII to prepare the plasmid 20,298-left arm-Ubc-CMV-nlsmtagBFP-bGHpA-right arm; (5) the expression cassette of EGFP-P2A-EGFP-F2A-EGFP-T2A-TK was inserted into the digested plasmid 20,298-left arm-Ubc-CMV-nlsmtagBFP-bGHpA-right arm with MfeI and EcoRI to prepare the plasmid PS676. To construct the plasmid PS829 **(**left arm-Ubc-DIO-mRuby3-P2A-mRuby3-F2A-mRuby3-T2A-TK-WPRE-hGHpA-CMV-nlsmtagBFP-bGHpA-right arm), the expression cassette of mRuby3-P2A-mRuby3-F2A-mRuby3-T2A-TK was inserted into the digested plasmid PS676 with FseI and SwaI to prepare the plasmid PS829. To construct the plasmid PS292 **(**left arm-Ubc-DsRed-SV40pA-right arm), the expression cassette of DsRed-SV40pA was inserted into the digested the plasmid PS676 with MfeI and AsiSI to prepare the plasmid PS292. All plasmids were confirmed by means of sequencing.

### Preparation of Recombinant viruses

PRV292, PRV676, and PRV829 were prepared by means of homologous recombination between a donor plasmid (PS292, PS676, and PS829) containing a left arm and a right arm and the genome of the PRV Bartha strain. First, BHK-21 cells were transfected with 2 µg of plasmid (PS292), and the transfected BHK-21 cells were infected with the PRV Bartha strain at a multiplicity of infection (MOI) of 1 at 6 h post-transfection (hpi), and then the medium was collected at 2 days post-infection (dpi). Next, the plaque assay was performed to purify the recombinant PRV292. Second, the BHK-21 cells were transfected separately with 2 µg of plasmid (PS676 or PS829), the transfected BHK-21 cells were infected with PRV292 (moi = 1) after 6 hpi, and the medium was collected at 2 dpi. Then, the plaque assay was performed to purify the recombinant PRV676 and PRV829.

Next, the purified PRV676 and PRV829 were separately added into the T75 flask containing BHK-21 cells with 90% confluency. After two days, the cell culture medium was collected and filtered with 0.22 μm filter, respectively. Then, they were centrifuged at 50,000 g for 2 h at 4 °C and repeat again. At last, the pellets were suspended in the cold PBS and stored at − 80 °C.

### Growth curve

The BHK-21 cells were infected with PRV676 and PRV829 at an MOI of 0.1. The supernatants were harvested at 12, 24, 36, 48, 60, and 72 hpi. Then, the viral titer of each sample was assessed using the plaque assay.

### Plaque assay

A plaque assay was performed as described in a previous study [16]. Briefly, 100 µL of the diluted sample was added to each well of 6-well plates containing BHK-21 cells, and then the plates were incubated at 37 °C with 5% CO_2_ for 1 h. Then, a first layer of agar was added into each well. After 48 hpi, a second layer of agar containing neutral red was added. The plaque number was determined after an additional 24 h of incubation. The viral titer was calculated as the number of plaque-forming units (PFU) per milliliter.

### Stereotaxic microinjection in mice brain

Animal experiments were performed using previously described methods [16, 21]. For testing the characteristics of these two tracers in the presence and absence of Cre in vivo, PRV676 (9.3 × 10^9^ PFU/ml, 300 nl) and PRV829 (1.8 × 10^9^ PFU/ml, 300 nl) were separately stereotaxically microinjected into the ventral tegmental area (VTA, AP, -3.4 mm, ML, + 0.4 mm, DV, -4.3 mm) of eight-week-old male C57BL/6J mice and DAT-Cre mice. For mapping the neural circuit of neurons expressing Cre, PRV676 (9.3 × 10^9^ PFU/ml, 300 nl) and PRV829 (4.5 × 10^9^ PFU/ml, 300 nl) were separately stereotaxically microinjected into the VTA region of DAT-Cre mice and the dorsolateral striatum (DLS, AP, + 0.86 mm, ML, + 2.3 mm, DV, -3.5 mm) of vGat-Cre mice. Then, mice were anesthetized with pentobarbital sodium (I.P., 80 mg/Kg, 1% in sterile saline) and placed in a stereotaxic apparatus (RWD, 68030, 68025). At the indicated time, mice were deeply anaesthetized with an overdose of pentobarbital sodium (100 mg/Kg) and were transcardially perfused with PBS followed by 4% paraformaldehyde solution (in PBS). The brains were removed and post-fixed overnight in 4% paraformaldehyde and dehydrated in 30% sucrose (in PBS) before being sectioned into 40 μm slices with freezing microtome (Leica CM1950). Imaging was performed using a confocal laser scanning microscope (ZEISS LSM900).

### Immunohistochemistry

For staining the NeuN, fixed slices were immunostained with the NeuN antibody (1:400, Abcam, ab177487) and amplified with the goat anti-rabbit secondary antibody (1:500, Jackson, #111-586-144). For staining the Cre, fixed slices were immunostained with the Cre antibody (1:400, Sigma, MAB3120) and amplified with the goat anti-mouse secondary antibody (1:500, Abcam, ab150116). Slices were imaged using the Leica TCS SP8 confocal microscope or the Olympus VS 120 slide scanning system.

## Results

### **Preparation of Cre-dependent PRV tracers expressing EGFP or mRuby3**

**Fig. 1:**
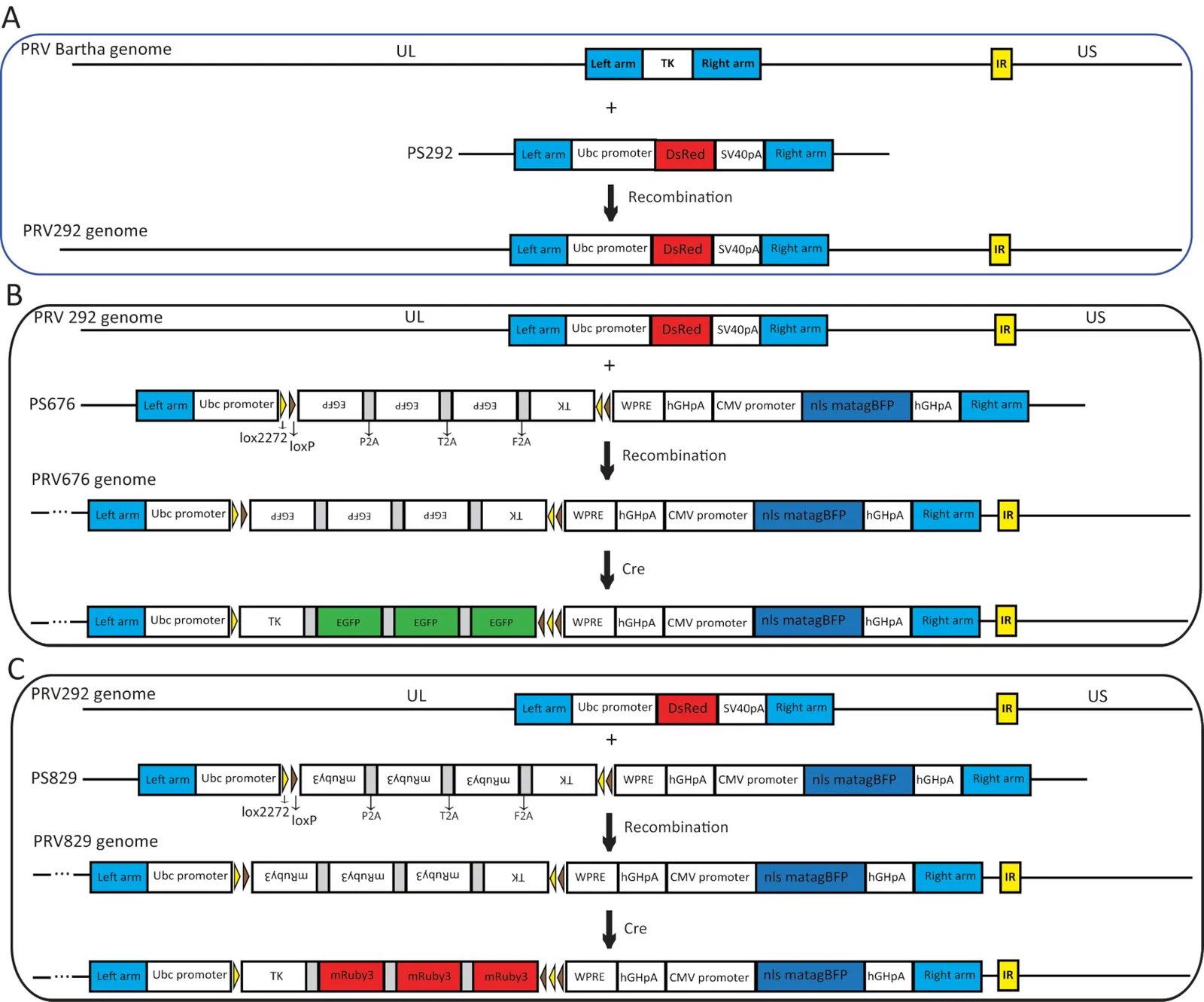
Schematic diagram of preparation of PRV tracers. (**A**). The PRV292 was prepared by means of homologous recombination between PS292 and the genome of PRV Bartha strain, which was used for preparing the PRV676 and PRV829 tracers. The TK gene was deleted from the genome of PRV292. The PRV676 and PRV829 were generated by using the strategy in (**B**) and (**C**), respectively. Briefly, the PS676 and PS829 were separately tranfected into BHK-21 cells, and then the PRV292 was added into the wells. If the Cre was present in neurons, the fluoresent protein and the TK protein were expressed, and then the progeny viruses were produced

Our previous work showed that the three copies of EGFP had the highest expression level when comparing one and six copies of EGFP with the same promoter [16]. Therefore, to produce the cre-dependent retrograde trans-multisynaptic PRV tracer, the Cre-dependent expression cassettes of three copies of EGFP (or mRuby3) and TK replaced the TK gene of PRV Bartha via homologous recombination (Fig. 1). First, to prevent the recombination of the TK gene between the donor plasmid (PS676 or PS829) and the viral genome, PRV292 was produced by means of homologous recombination between the donor plasmid PS292 and the PRV Bartha strain (Fig. 1A). In PRV292, the TK gene was deleted. Secondly, PRV676 and PRV829 were separately prepared by inserting the Cre-dependent expression cassettes of three copies of EGFP (or mRuby3) and TK into the genome of PRV292 (Fig. 1B and C).

**Fig. 2:**
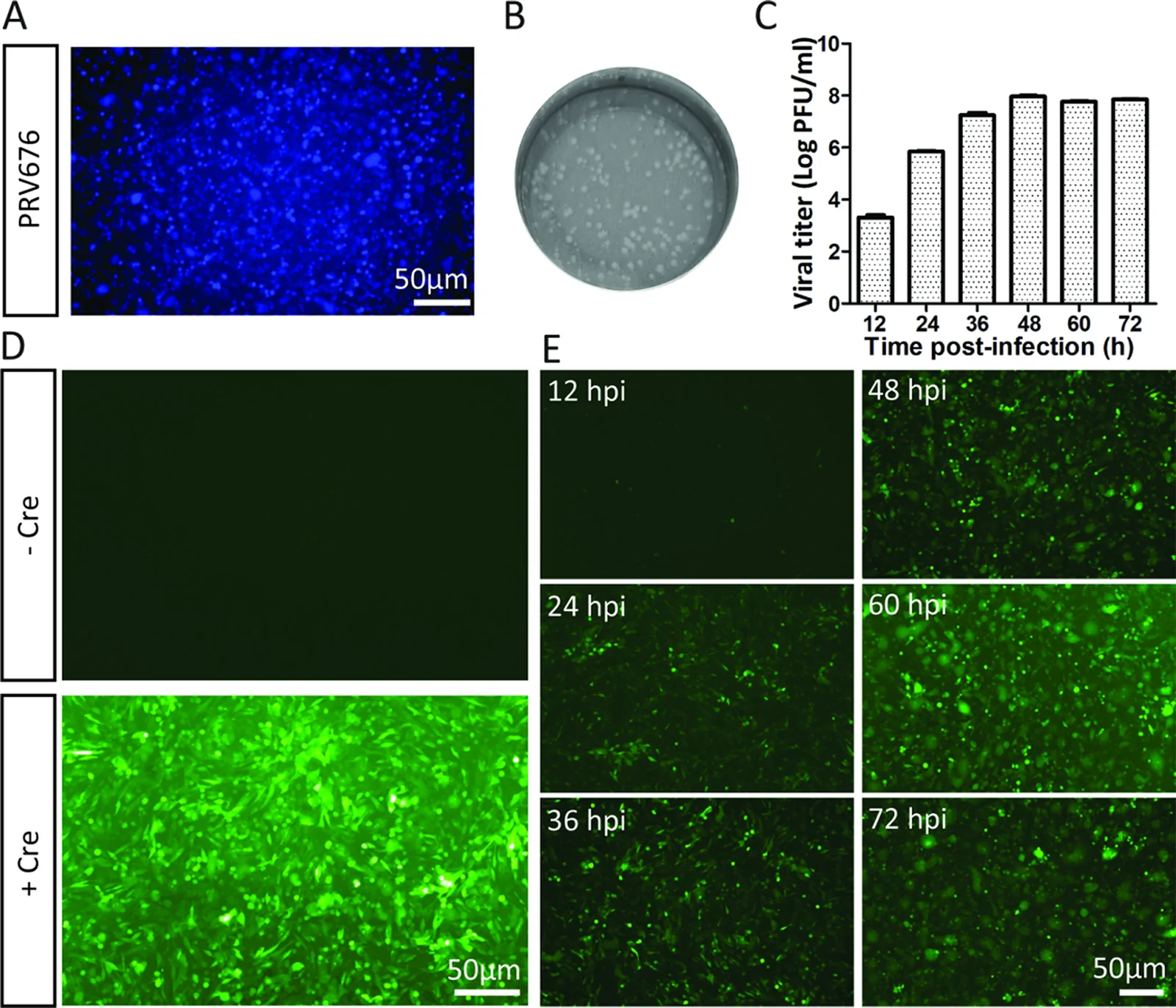
The characteristic of PRV676 in vitro. (**A**) The blue fluorescent protein (BFP) was expressed when the PRV676 infected the BHK-21 cells, which provided a direct signal for virus production in vitro. (**B**) The PRV676 can infect BHK-21 cells and produce plaque. (**C**) The growth curve of PRV676. The BHK-21 cells were infected by the PRV676 at an MOI of 0.1. The supernatant was collected at 12, 24, 36, 48, 60, and 72 hpi, and the titers of these samples were determined by using plaque assay. (**D**) The EGFP was expressed under the control of Cre. (**E**) The time course of EGFP expression and virus production in BHK-21 cells expressing Cre. The EGFP signals were observed at 12, 24, 36, 48, 60, and 72 hpi

**Fig. 3:**
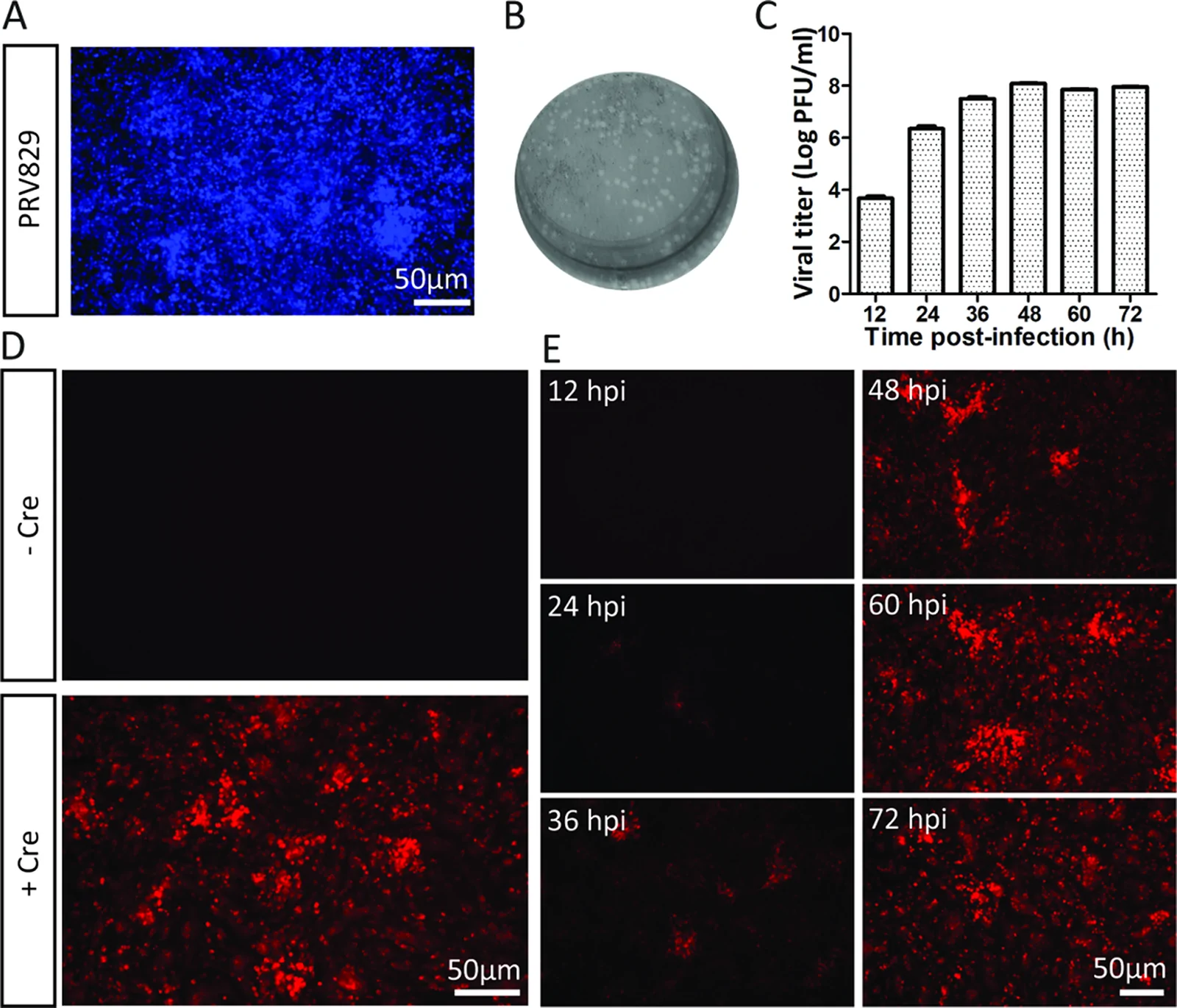
The characteristic of PRV829 in vitro. (**A**) The blue fluorescent protein (BFP) was expressed when the PRV829 infected the BHK-21 cells, which provided a direct signal for virus production in vitro. (**B**) The PRV829 can infect BHK-21 cells and produce plaque. (**C**) The growth curve of PRV829. The BHK-21 cells were infected by the PRV829 at an MOI of 0.1. The supernatant was collected at 12, 24, 36, 48, 60, and 72 hpi, and the titers of these samples were determined by using plaque assay. (**D**) The mRuby3 was expressed under the control of Cre. (**E**) The time course of mRuby3 expression and virus production in BHK-21 cells expressing Cre. The mRuby3 signals were observed at 12, 24, 36, 48, 60, and 72 hpi

The purified recombinant PRV676 and PRV829 can express a blue fluorescent protein (Figs. 2A and 3A) and produce similar plaque sizes (Figs. 2B and 3B). In addition, the growth curves of PRV676 and PRV829 indicated that the amounts of tracers increased from 12 to 48 hpi and were then reduced (Figs. 2C and 3C). These two tracers could separately express EGFP and mRuby3 when the Cre was expressed in BHK-21-Cre cells (Figs. 2D and 3D) and the density of fluorescent signals was increased with the time course (Figs. 2E and 3E). Collectively, these results indicate that PRV676 and PRV829 were prepared successfully, and separately expressed EGPF and mRuby3 in the presence of Cre.

### PRV676 and PRV829 Cre-dependently express fluorescent protein and produce virus progeny in vivo

**Fig. 4:**
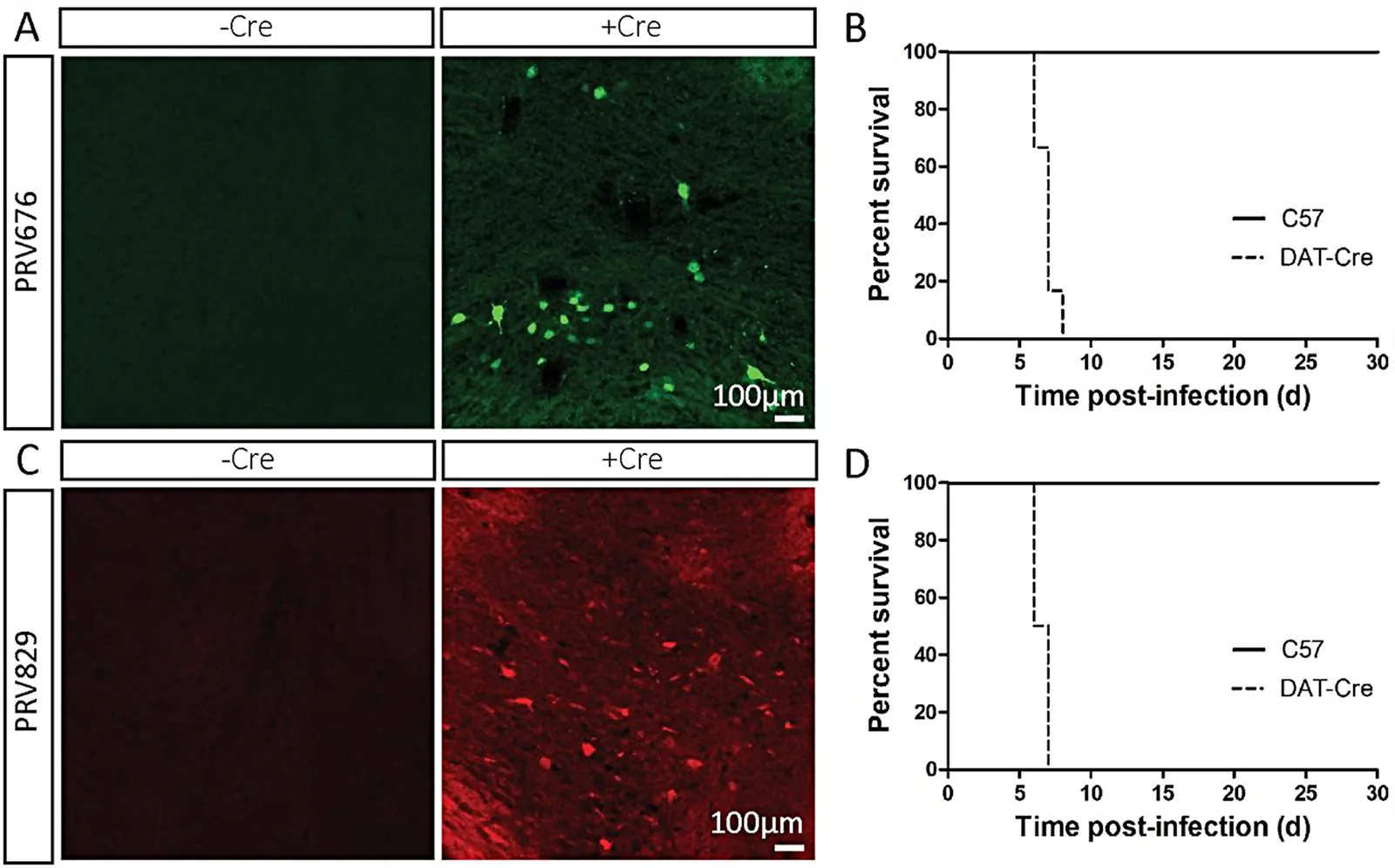
PRV676 and PRV829 Cre-dependently express fluorescent protein and produce progeny viruses in brain. (**A**) and (**B**) The PRV676 (9.3 × 10^9^ PFU/ml, 300 nl) was separately injected into the VTA of the C57BL/6J mice and DAT-Cre mice. (**A**). The C57BL/6J mice infected with the PRV676 survived through the experimental stage, while DAT-Cre mice infected by PRV676 was dead by 6 dpi (**B**). (**C**) and (**D**) The PRV829 (1.8 × 10^9^ PFU/ml, 300 nl) was separately injected into the VTA of the C57BL/6J mice and DAT-Cre mice. The mRuby3 was only observed in neurons expressing Cre (**C**). The C57BL/6J mice infected with the PRV829 survived through the experimental stage, while DAT-Cre mice infected by the PRV829 was dead by 6 dpi (**D**)

Based on the results shown in Fig. 2D, we can infer that PRV676 and PRV829 strictly Cre-dependently express EGFP and mRuby3 in vitro. Here, these two tracers were separately stereotaxically microinjected into the VTA of the C57BL/6J mouse brain and DAT-cre mouse brain to test whether they show the strict Cre-dependent expression of EGFP and mRuby3. Indeed, no EGFP and mRuby3 were observed in the injection site of the C57BL/6J mice brain, while they were observed in the injection site of the DAT-Cre mouse brain (Fig. 4A and C). In addition, the C57BL/6J mice infected with these two tracers survived through the experimental stage (observed for one month), but the DAT-Cre mice were dead by 6 dpi (Fig. 4B and D). These results indicate that these two tracers strictly Cre-dependently expressed EGFP and mRuby3 and produced virus progeny in vivo.

### The tracers can map the multilevel input neural circuit of specific neurons expressing Cre

**Fig. 5:**
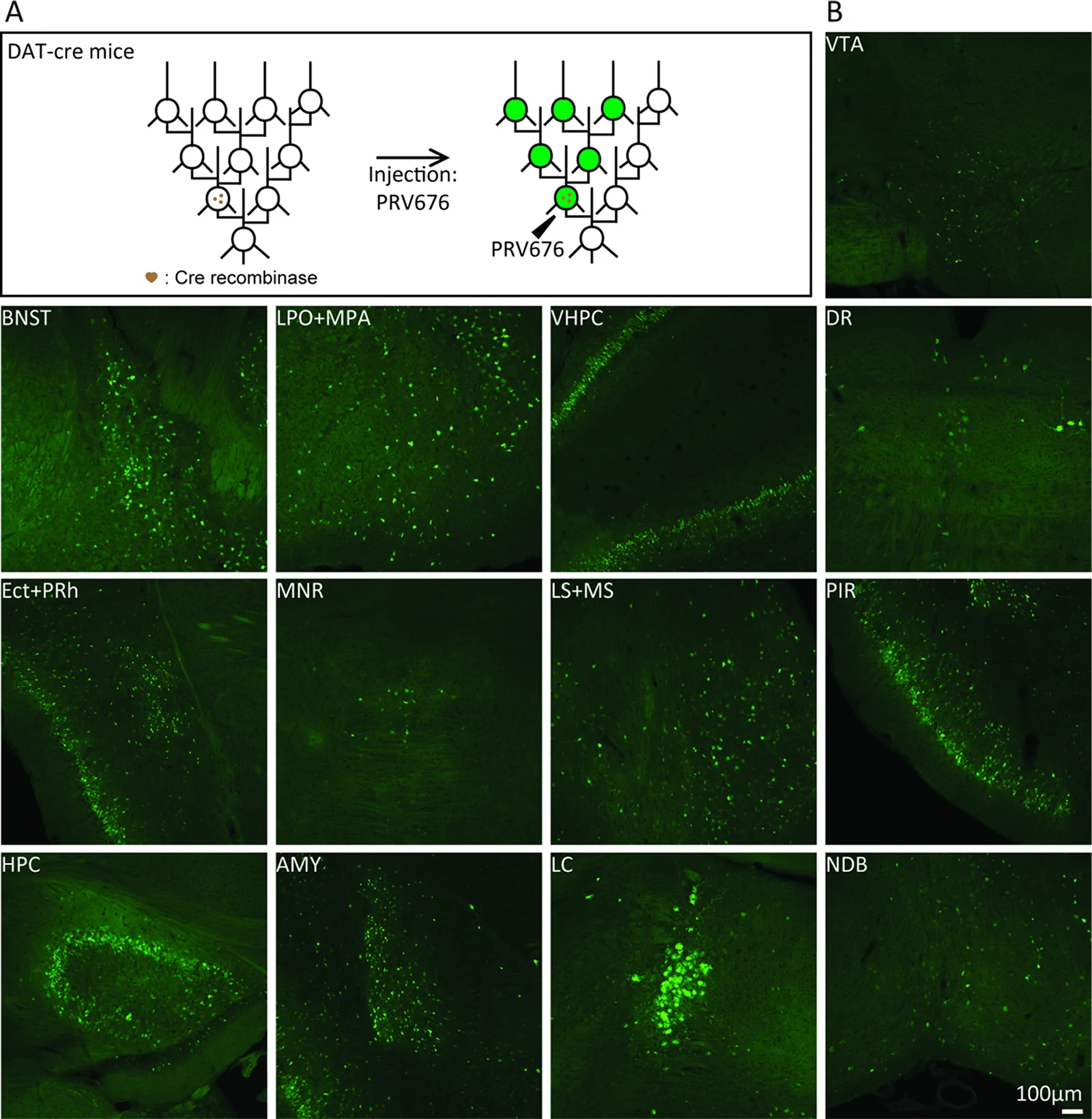
PRV676 can map the multilevel inputs of neurons expressing Cre in VTA of DAT-Cre mice. (**A**) The schematic diagram of PRV676 injection and retrograde trans-multisynaptic spreading. (**B**) The PRV676 (9.3 × 10^9^ PFU/ml, 300 nl) was separately injected into the VTA of the DAT-Cre mice. The brains were treated and cut at 7 dpi. The EGFP was observed in various brain regions. BNST, bed nucleus of the stria terminalis; LPO, lateral preoptic area; MPA, medial preoptic area; VHPC, ventral hippocampus; DR, dorsal raphe nucleus; Ect, ectorhinal cortex; PRh, perirhinal cortex; MNR, median raphe nucleus; LS, lateral septal nucleus; MS, medial septal nucleus; PIR, piriform cortex; HPC, hippocampus; AMY, amygdala; LC, locus coeruleus; NDB, nucleus of the diagonal band

**Fig. 6:**
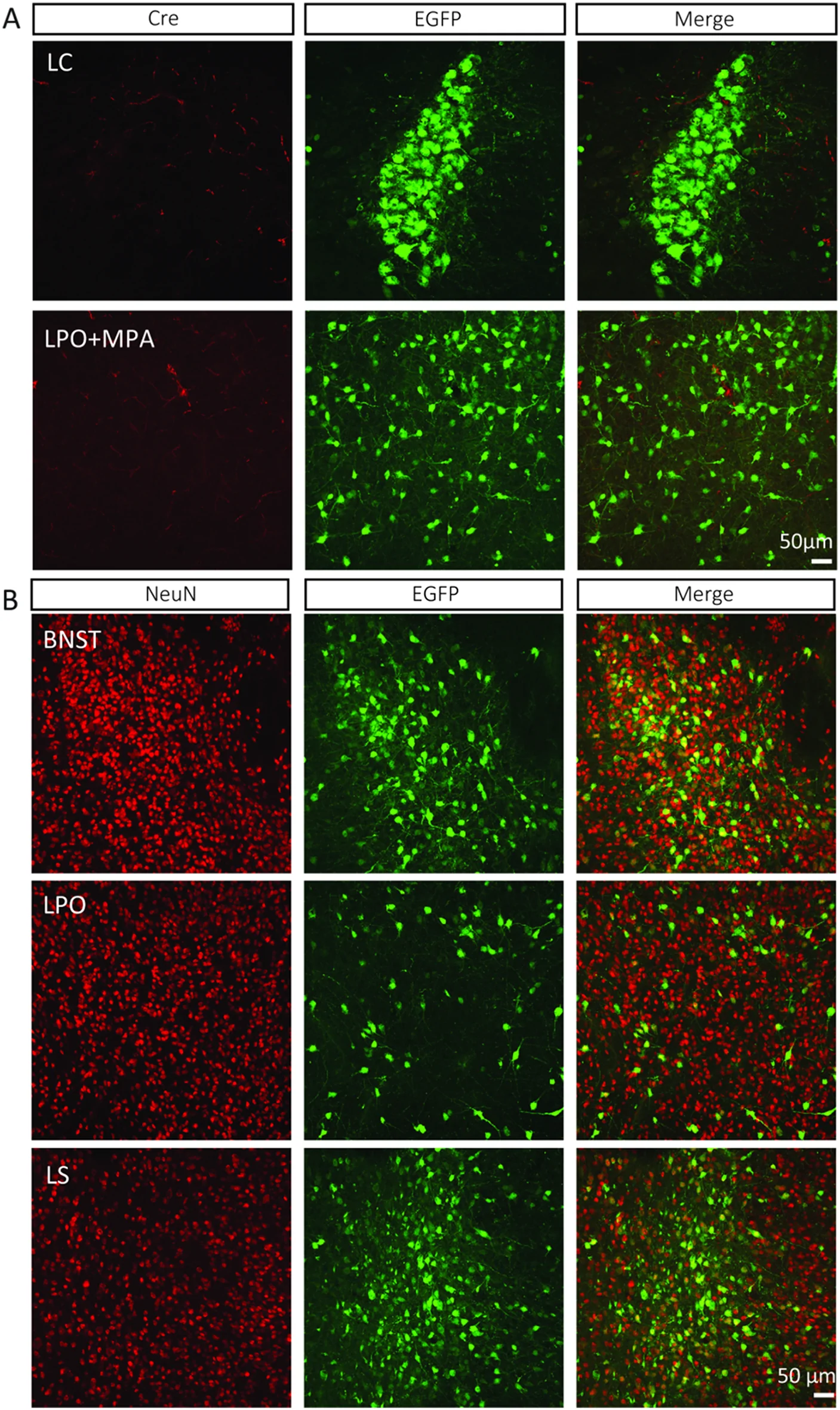
The infection of PRV676 on brain sections can not impede the molecular analysis. The PRV676 (9.3 × 10^9^ PFU/ml, 300 nl) was injected into the VTA of the DAT-Cre mice. The brains were treated and cut at 7 dpi. The Cre (**A**) and NeuN (**B**) on brain sections were immunostained with Cre antibody and NeuN antibody, respectively. BNST, bed nucleus of the stria terminalis; LPO, lateral preoptic area; LS, lateral septal nucleus; LC, locus coeruleus; MPA, medial preoptic area

**Fig. 7:**
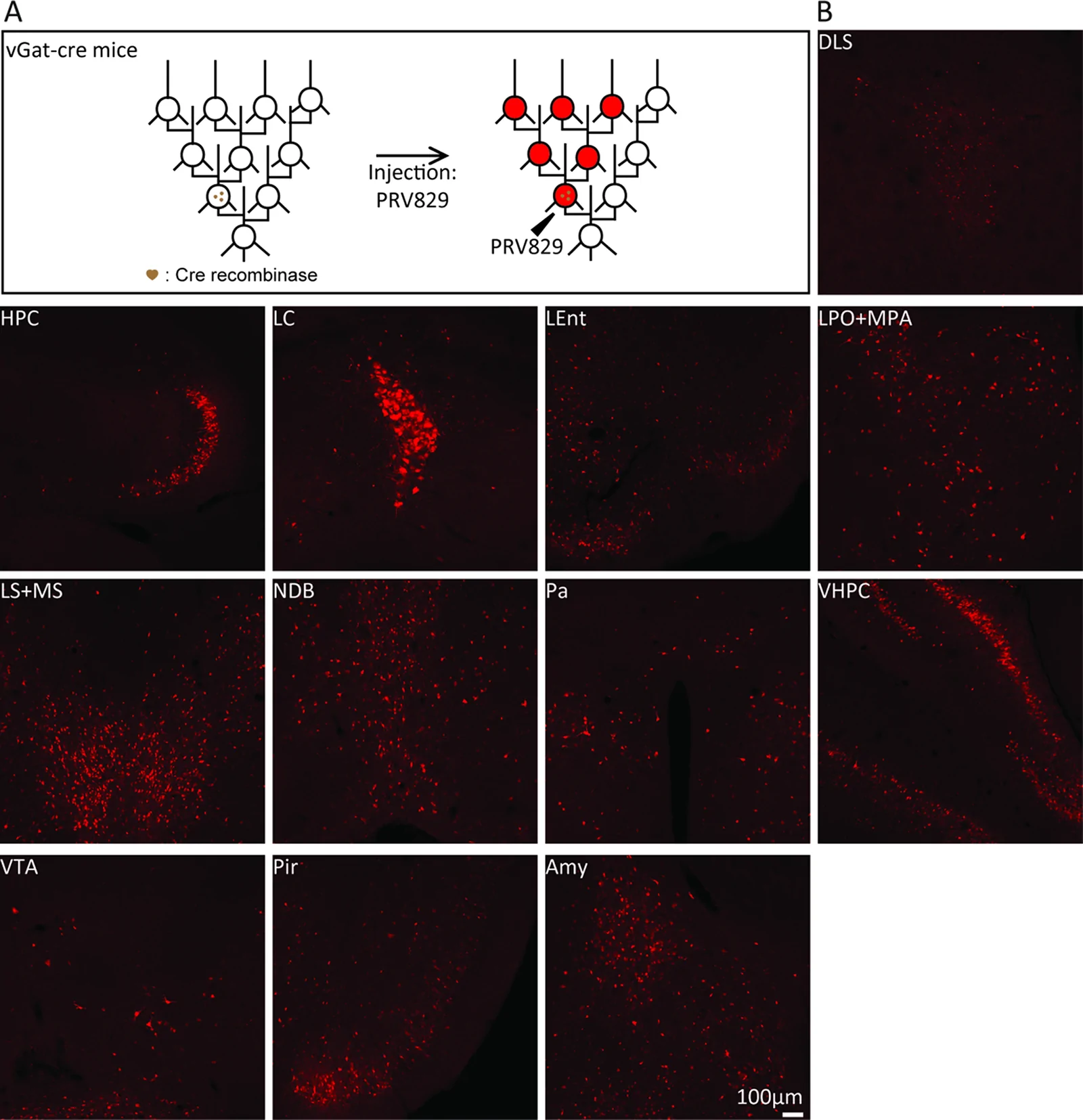
PRV829 can map the multilevel inputs of neurons expressing Cre in DLS of vGAT-Cre mice. (**A**) The schematic diagram of PRV829 injection and retrograde trans-multisynaptic spreading. (**B**) The PRV829 (4.5 × 10^9^ PFU/ml, 300 nl) was separately injected into the DLS of the vGAT-Cre mice. The brains were treated and cut at 6 dpi. The mRuby3 was observed in various brain regions. HPC, hippocampus; LC, locus coeruleus; LEnt, lateral entorhinal cortex; Pa paraventricular hypothalamic nucleus; LS, lateral septal nucleus; MS, medial septal nucleus; NDB, nucleus of the diagonal band; VTA, ventral tegmental area; LPO, lateral preoptic area; MPA, medial preoptic area; VHPC, ventral hippocampus; AMY, amygdala; PIR, piriform cortex

The above results demonstrate that the PRV676 and PRV829 can Cre-dependently express a fluorescent protein and produce virus progeny in vitro and in vivo. Here, we made an attempt to test the abilities of these two tracers to map the multilevel inputs of a specific neuronal subpopulation expressing Cre. For PRV676, the tracer was injected into the VTA region of the DAT-Cre mouse brain, and EGFP-positive neurons were observed in VTA, BNST, LPO, MPA, VHPC, DR, Ect, PRh, MNR, LS, MS, PIR, HPC, AMY, LC, and NDB (Fig. 5). Among them, several brain sections containing LC, LPO, and MPA were selected to perform immunostaining with Cre antibody, and the inputs signals (EGFP) were absent in the Cre-positive neurons (Fig. 6A), which demonstrate that the PRV676 can undergo retrograde transsynaptic spread within neural circuit. In addition, the EGFP signals in injection site VTA were weak and sparse, which might be caused by the cytotoxicity of PRV tracer. To confirm whether the cytotoxicity of PRV could have an impact on the molecular identity of input neurons, the general neuronal marker NeuN was immunostained on the brain sections containing infected neurons, and the NeuN signals were colabeled with the EGFP signals in the neurons containing virus (Fig. 6B). Furthermore, the ratio of the EGFP-positive neurons among the NeuN-positive neurons in BNST, LPO, and LS was presented in Suppl. Figure 1. These results demonstrate that the cytotoxicity of PRV does not impede the molecular analysis and neural circuit labeling. For PRV829, the tracer was injected into the DLS region of the vGat-cre mouse brain, and mRuby3-positive neurons were found in DLS, HPC, LC, LEnt, Pa, LS, MS, NDB, VTA, LPO, MPA, PIR, AMY, and VHPC (Fig. 7). These results indicate that PRV676 and PRV829 can be used to depict the multilevel inputs of a specific subpopulation of neurons expressing Cre.

## Discussion

Various strains of the Pseudorabies virus were isolated and characterized in previous studies [22]. Among them, the PRV Bartha strain can spread in a trans-synaptic and retrograde manner in the neural circuit due to its genome lacking the US9 gene [23]. Here, we constructed two new retrograde trans-multisynaptic tracers (PRV676 and PRV829), which can be used to map the neural circuit of a specific neuronal subpopulation expressing Cre. In these tracers, the TK gene was replaced with a Cre-dependent 3×EGFP-TK (PRV676) or 3×mRuby3-TK (PRV829) expression cassette under the control of the Ubc promoter (Fig. 1b and c). In our previous work, we demonstrated that the PRV, as a vector, expresses a low level of the inserted gene [16]. To visualize the neural circuit, greater fluorescent protein expression is needed. Interestingly, the levels of expression can be ranked as follows: three copies EGFP > six copies EGFP > one copy of EGFP [16]. Therefore, we inserted three copies of EGFP or mRuby3 into the genome of the PRV Bartha strain to develop these tracers (Fig. 1b and c).

The cytotoxicity of neural circuit tracers is a common problem that has limited their application [2]. Many measures have been taken to reduce the cytotoxicity of other tracers, such as the rabies virus [8, 10, 24–26], vesicular stomatitis virus [27], and herpes simplex virus [15]. Although PRV Bartha is an attenuated vaccine strain, it can still kill mice by about 3 dpi when injected into the brain. Based on the results of our study, the C57BL/6J mice were not killed by these two tracers as the viruses could not propagate in neurons lacking Cre. These tracers can map the multisynaptic inputs of a specific neuronal subpopulation expressing Cre, and the infected transgenic mice died after about 6 dpi. To obtain more fluorescent signals on the brain section, it is advisable to prepare the brain sections when the animal exhibits severe symptoms, yet before it reaches the terminal stage of death. Therefore, like other tracers, the Cre-dependent retrograde trans-multisynaptic PRV tracers also have high cytotoxicity. Future works should be focused on reducing their cytotoxicity. Furthermore, determining the time course of infection is important to understand the speed of transsynaptic spread, which is helpful for how to use these tracers.

In addition, both PRV and RV tracers can be used to map the input neural circuits. However, there are notable differences between them. (1) The genome of PRV is DNA. It can be used to label the multilevel and monolevel inputs into the neural circuit of a specific neuronal subpopulation. Moreover, PRV Bartha strain does not infect the neurons of primate, so it has good safety. (2) The genome of RV is RNA. It can be used to map the multilevel and monolevel inputs in the neural circuit. Importantly, it can also be used to map the input neural circuit of primate due to it can infect the neurons of primate and spread in the neural circuit. However, more attention should be paid to safety when using this tool. (3) PRV and RV might have discrepant neurotropism within certain brain regions [28]. Therefore, both these two tracers are important for depicting the neural circuits.

Collectively, our work provides two new Cre-dependent retrograde trans-multisynaptic tracers, which will be productive in the context of neuroscience research.

Supplementary Fig. 1. The ratio of the EGFP-positive neurons among the NeuN-positive neurons in BNST, LPO, and LS. The PRV676 (9.3 × 10^9^ PFU/ml, 300 nl) was injected into the VTA of the DAT-Cre mice (*n* = 3). The brains were treated and cut at 7 dpi. The NeuN on brain sections were immunostained with NeuN antibody. To determine the ratio of the EGFP-positive neurons among the NeuN-positive neurons in BNST, LPO, and LS. Brain slices from each animal were selected for data analyzing. The ratio of the EGFP-positive neurons among the NeuN-positive neurons was calculated as the number of the EGFP-positive neurons divided by the number of NeuN-positive neurons in BNST, LPO, and LS, respectively. Error bars indicate mean ± SEM.

## Electronic Supplementary Material

Below is the link to the electronic supplementary material.

Supplementary Material 1

## Data Availability

No datasets were generated or analysed during the current study.
