# Correction to: Development of Cre-dependent retrograde trans-multisynaptic tracer based on pseudorabies virus bartha strain

**DOI:** 10.1186/s13041-025-01241-7

**Published:** 2026-10-30

**Authors:** Hu You, Wang Qinghan, Sun Kangyixin, Yang Jia, Xu Fuqiang, Jia Fan

**Affiliations:** 1 Provincial Key Laboratory of Brain Connectome and Behavior, CAS Key Laboratory of Brain Connectome and Manipulation, the Brain Cognition and Brain Disease Institute, Translational Research Center for the Nervous System, Shenzhen Institutes of Advance Technology, Chinese Academy of Sciences, Shenzhen-Hong Kong Institute of Brain Science-Shenzhen Fundamental Research Institutions, Shenzhen, 518055 China; 2 NMPA Key Laboratory for Research and Evaluation of Viral Vector Technology in Cell and Gene Therapy Medicinal Products, Key Laboratory of Quality Control Technology for Virus- Based Therapeutics, Shenzhen Key Laboratory of Viral Vectors for Biomedicine, Shenzhen Institutes of Advance Technology, Guangdong Provincial Medical Products Administration, Chinese Academy of Sciences, Shenzhen, 518055 China; 3 Wuhan National Laboratory for Optoelectronics, Huazhong University of Science and Technology, Wuhan, 430074 China; 4 University of Chinese Academy of Sciences, Beijing, 100049 China; 5 Faculty of Life and Health Sciences, Shenzhen University of Advanced Technology, Shenzhen, 518106 China

**Correction to: Mol Brain 18, 33 (2025).**

**https://doi.org/10.1186/s13041-025-01204-y**

Following publication of the original article [1], the authors identified that the green fluorecence gene EGFP that was used in their study contained an error by indicating the other green fluorecence gene EGFP Fig. 1.

The incorrect Fig. 1:

**Fig. 1:**
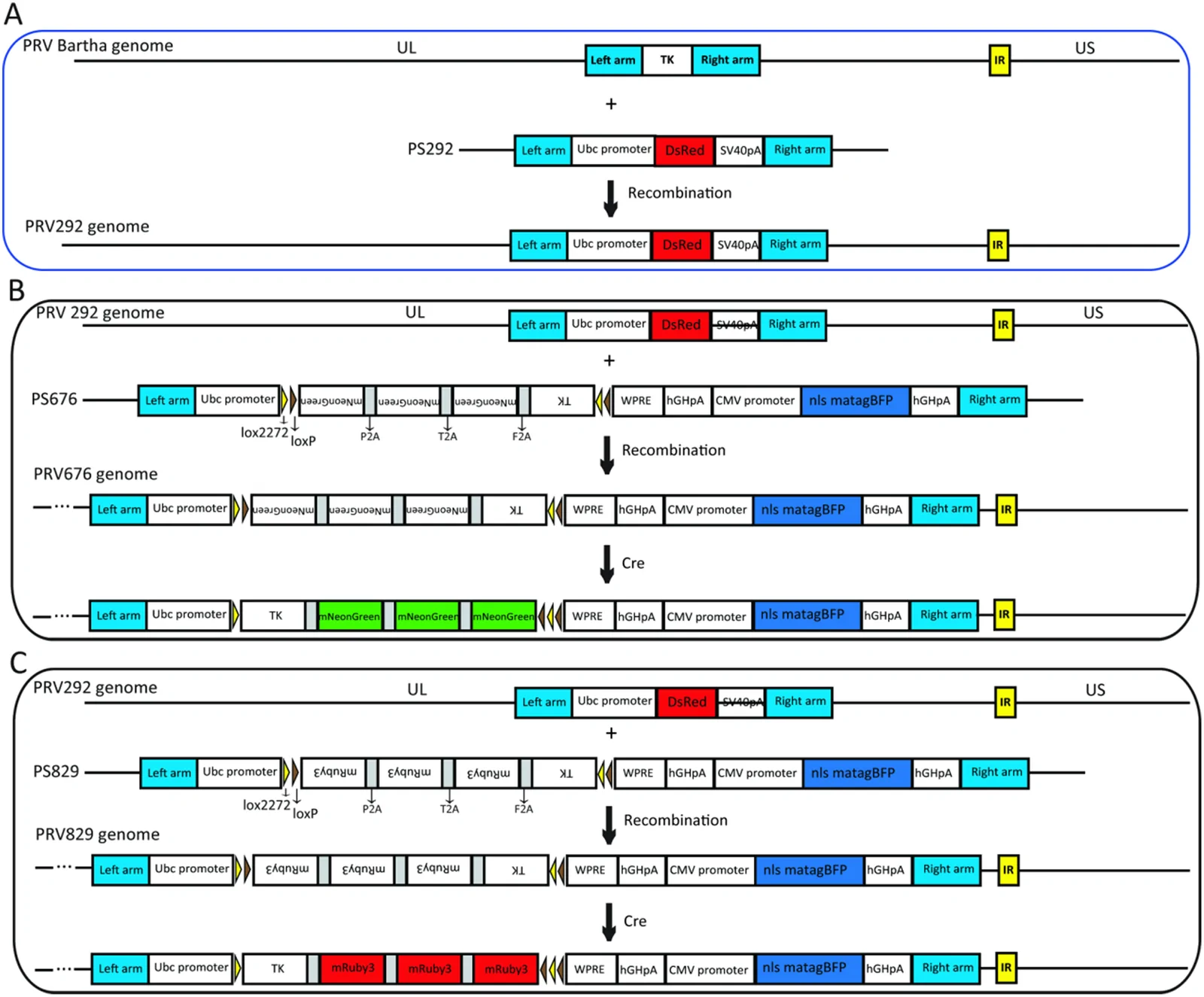
Schematic diagram of preparation of PRV tracers. (**A**). The PRV292 was prepared by means of homologous recombination between PS292 and the genome of PRV Bartha strain, which was used for preparing the PRV676 and PRV829 tracers. The TK gene was deleted from the genome of PRV292. The PRV676 and PRV829 were generated by using the strategy in (**B**) and (**C**), respectively. Briefly, the PS676 and PS829 were separately tranfected into BHK-21 cells, and then the PRV292 was added into the wells. If the Cre was present in neurons, the fluoresent protein and the TK protein were expressed, and then the progeny viruses were produced

The correct Fig. 1:

**Fig. 1:**
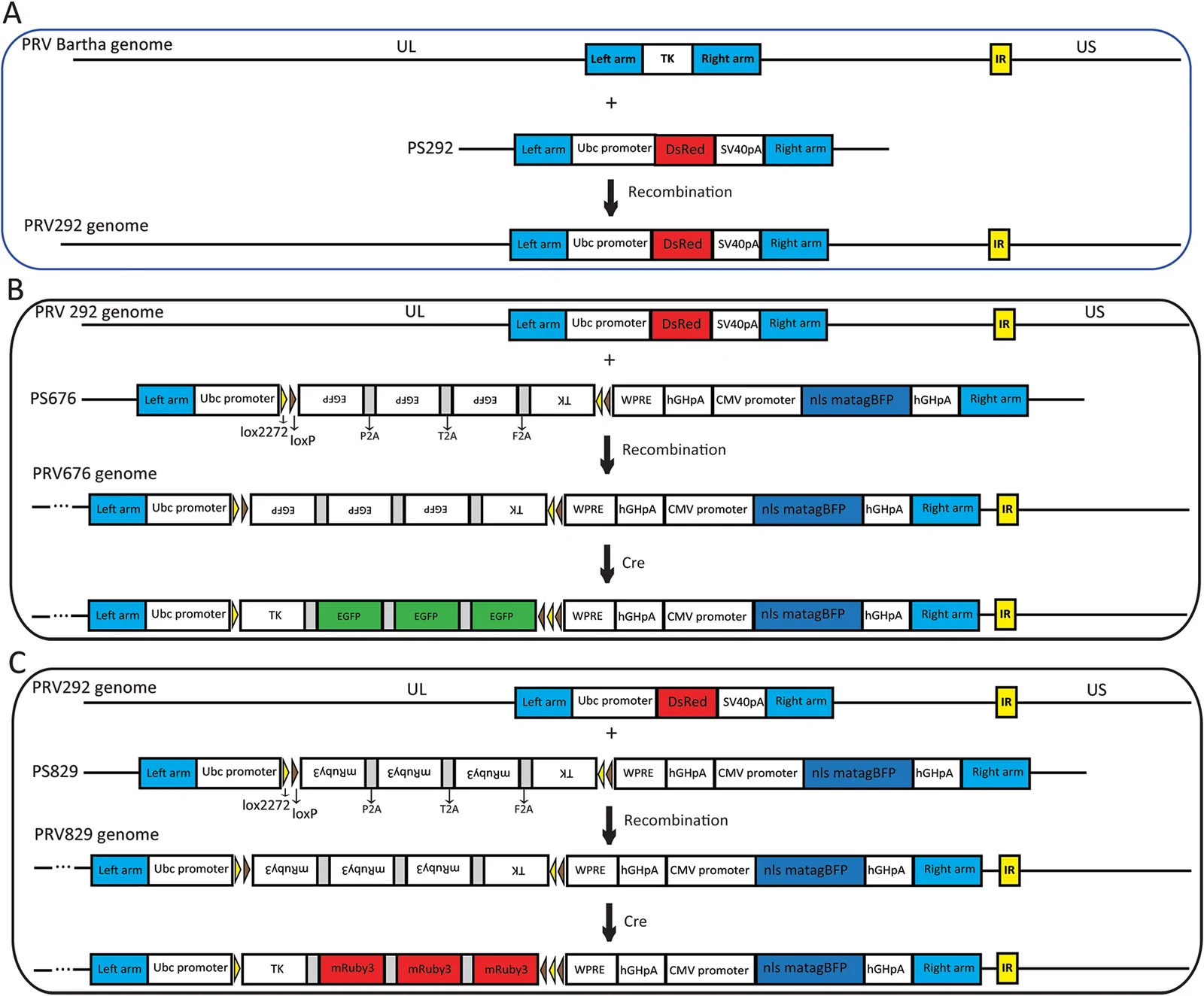
Schematic diagram of preparation of PRV tracers. (**A**). The PRV292 was prepared by means of homologous recombination between PS292 and the genome of PRV Bartha strain, which was used for preparing the PRV676 and PRV829 tracers. The TK gene was deleted from the genome of PRV292. The PRV676 and PRV829 were generated by using the strategy in (**B**) and (**C**), respectively. Briefly, the PS676 and PS829 were separately tranfected into BHK-21 cells, and then the PRV292 was added into the wells. If the Cre was present in neurons, the fluoresent protein and the TK protein were expressed, and then the progeny viruses were produced

Figure 1 has been updated and the original article [1] has been corrected.
