# Supplementary material for: Development of Cre-dependent retrograde trans-multisynaptic tracer based on pseudorabies virus bartha strain
Source: Mol Brain. 2025 Apr 14;18:33. doi: 10.1186/s13041-025-01204-y (PMC11995500; doi:10.1186/s13041-025-01204-y)

Supplementary Figure 1. The ratio of the EGFP-positive neurons among the NeuN-positive neurons in BNST, LPO, and LS. The PRV676 (9.3×10^9^ PFU/ml, 300 nl) was injected into the VTA of the DAT-Cre mice (n=3). The brains were treated and cut at 7 dpi. The NeuN on brain sections were immunostained with NeuN antibody. To determine the ratio of the EGFP-positive neurons among the NeuN-positive neurons in BNST, LPO, and LS. Brain slices from each animal were selected for data analyzing. The ratio of the EGFP-positive neurons among the NeuN-positive neurons was calculated as the number of the EGFP-positive neurons divided by the number of NeuN-positive neurons in BNST, LPO, and LS, respectively. Error bars indicate mean ± SEM.


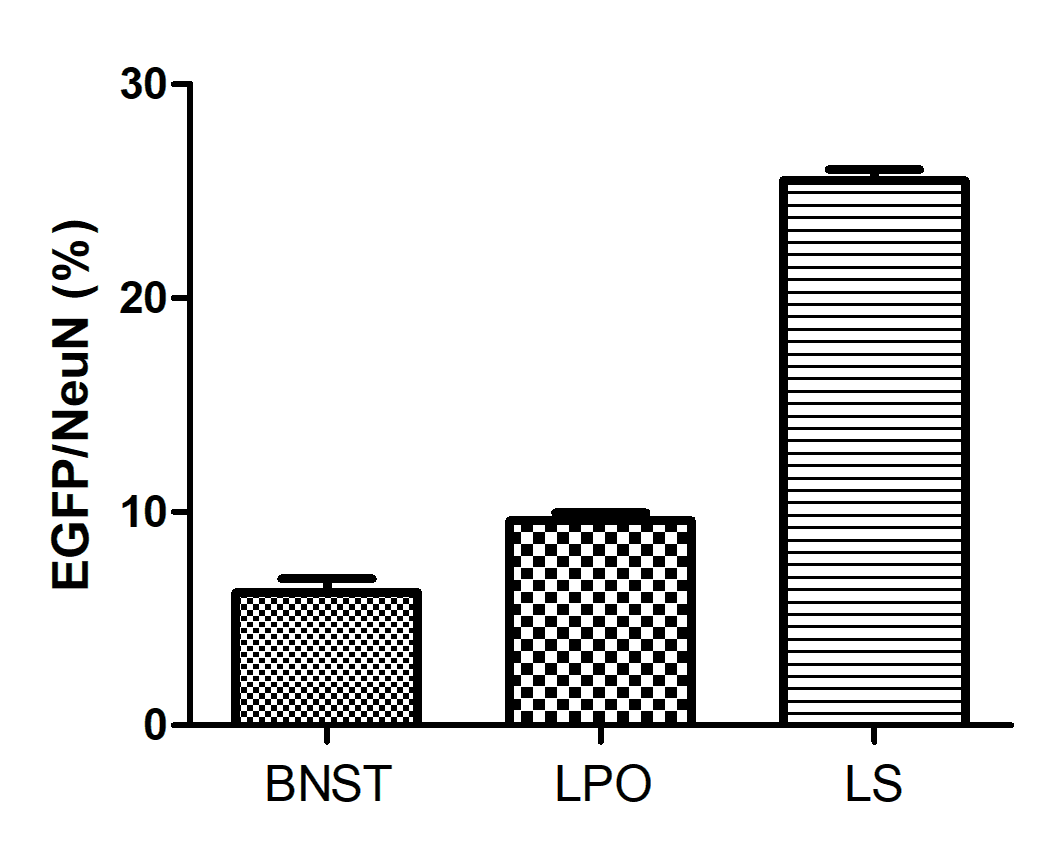

Supplement: Supplementary file 1 — Supplementary Material 1 [file 13041_2025_1204_MOESM1_ESM.docx]
